# Supplementary material for: Precise prediction of phase-separation key residues by machine learning
Source: Nat Commun. 2024 Mar 26;15:2662. doi: 10.1038/s41467-024-46901-9 (PMC10965946; doi:10.1038/s41467-024-46901-9)
Supplement: Supplementary file 1 — Supplementary Information [file 41467_2024_46901_MOESM1_ESM.pdf]

Precise prediction of phase-separation key residues by machine learning

Jun Sun[1,2,3,4,5,9], Jiale Qu[3,4,5,9], Cai Zhao[3,4,5,9], Xinyao Zhang[3,4,5], Xinyu Liu[3,4,5], Jia Wang[3,4,5,6], Chao Wei[3,4,5], Xinyi Liu[3,4,5], Mulan Wang[3,4,5], Pengguihang Zeng[3,4,5], Xiuxiao Tang[3,4,5], Xiaoru Ling[3,4,5], Li Qing[3,4,5], Shaoshuai Jiang[3,4,5], Jiahao Chen[3,4,5], Tara S. R. Chen[7], Yalan Kuang[1,2], Jinhang Gao[1,2], Xiaoxi Zeng[1,2], Dongfeng Huang[7], Yong Yuan[1,2]✉, Lili Fan[8]✉, Haopeng Yu[1,2]✉, Junjun Ding[1,2,3,4,5,7]✉

[1]Department of Thoracic Surgery and West China Biomedical Big Data Center, West China Hospital, Sichuan University, Chengdu 610041, China.

[2]Med-X Center for Informatics, Sichuan University, Chengdu 610041, China.

[3]RNA Biomedical Institute, Sun Yat-sen Memorial Hospital, Zhongshan School of Medicine, Sun Yat-sen University, Guangzhou, Guangdong, China.

[4]Advanced Medical Technology Center, The First Affiliated Hospital, Zhongshan School of Medicine, Sun Yat-sen University, Guangzhou, Guangdong, China.

[5]Center for Stem Cell Biology and Tissue Engineering, Key Laboratory for Stem Cells and Tissue Engineering, Ministry of Education, Zhongshan School of Medicine, Sun Yat-sen University, Guangzhou, Guangdong, China.

[6]GMU-GIBH Joint School of Life Sciences, Guangzhou Medical University, Guangzhou 511436, China

[7]Department of Rehabilitation Medicine, The Seventh Affiliated Hospital, Sun Yat-Sen University, Shenzhen, Guangdong, 518107, China.

[8]Guangzhou Key Laboratory of Formula-Pattern of Traditional Chinese Medicine, School of Traditional Chinese Medicine, Jinan University, Guangzhou, Guangdong, China

[9]These authors contributed equally to the work: Jun Sun, Jiale Qu, Cai Zhao.

✉e-mail: dingjunj@mail.sysu.edu.cn; yuhaopeng@wchscu.cn; fanlili@jnu.edu.cn; yongyuan@scu.edu.cn

SUPPLEMENTARY FIGURES AND LEGENDS

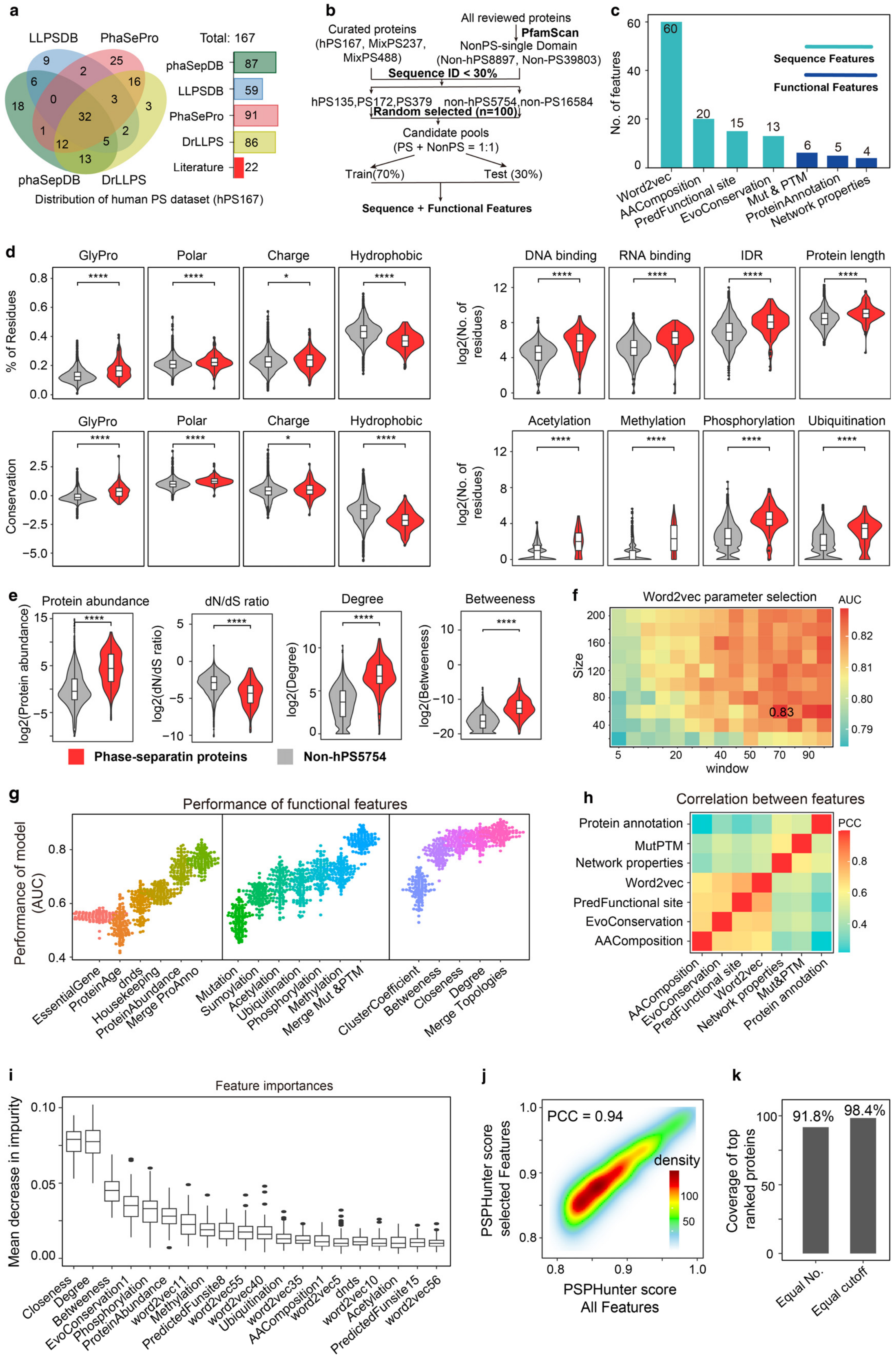

Supplementary Fig. 1 | The selected features provide information to hunt phase-separating proteins for the establishment of PSPHunter. **a**, Distribution of data sources within the human PS dataset (hPS167). **b**, Flowchart detailing the establishment process of the PSPHunter algorithm. **c,** The number of different types of features. **d-e**, Violin plots comparing the sequence and functional features between phase-separating proteins and NonhPS5754. (one-sided Wilcoxon test, n.s., no significance, ***P < 0.001, ****P < 0.0001). **f**, Parameter selection for the Word2Vec Method: In this context, 'window' refers to the maximum distance between the current and predicted words, while 'size' denotes the dimensionality of word vectors. **g,** Performance of individual functional features: Each panel represents the performance of a single functional feature, with the rightmost segment indicating the combined effect of that type of feature. **h**, Correlation analysis of various features based on their phase separation capacities in the cross-validation datasets. **i,** Distribution of importance scores for the top 20 features. Feature importances were obtained using the fitted attribute 'feature_importances_' from the scikit-learn package. **j,** Correlation between PSPHunter scores obtained from the model using all features and the model employing the selected features. It is noteworthy that we selected 898 phase-separating proteins using all features. **k**, Coverage comparison between proteins identified as phase-separating using all features and those identified using selected features. In relation to the selected feature model, when proteins are chosen based on their predictive probabilities ranking among the top, 91.8% of proteins are covered. Similarly, when the same threshold (0.82) is applied for the selected feature model, 98.4% of proteins are encompassed.

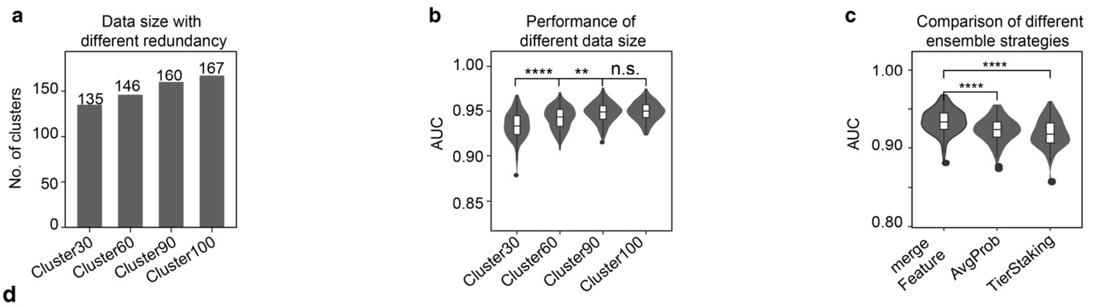

d

Method	Model	Organism	Dataset	Features
PLAAC	hidden Markov	S. cerevisiae	4/28	Prion-like domain
DDX4-likes	Statistical frequency	46 species	68	Sequence composition and residue spacing similarity to DDX4
CatGRANULE	Granule propensity	S. cerevisiae	120	Sequence length, R/G/F content, and amino acid propensity for nucleic acid binding and disorder
R + Y	Mean-field	Homo sapiens	-	Number of arginine and tyrosine residues
Pscore	statistical scoring	3 species	11	Expected numbers of long-range planar sp2 pi-pi contacts
CRAPome	-	Homo sapiens	-	Frequency at which each human protein is identified by affinity purification mass spectrometry negative controls
LARKs	-	Homo sapiens	-	Energetic compatibility with aromatic-rich kinked amyloid structures
PhaSePred	XGBoost	11 species	237	Hydro, FCR, IDR, LCR, Pscore, PLAAC, catGRA, DeepCoil, Phos, IF
PSPredictor	GBDT	74 species	353	word2vec, LQL
PSPHunter	Randomforest	Homo sapiens	167	Sequence feature: AAComposition, EvoConservation, PredFunctional site, Word2vec; Functional feature: Network properties, Mut&PTM, Protein annotation

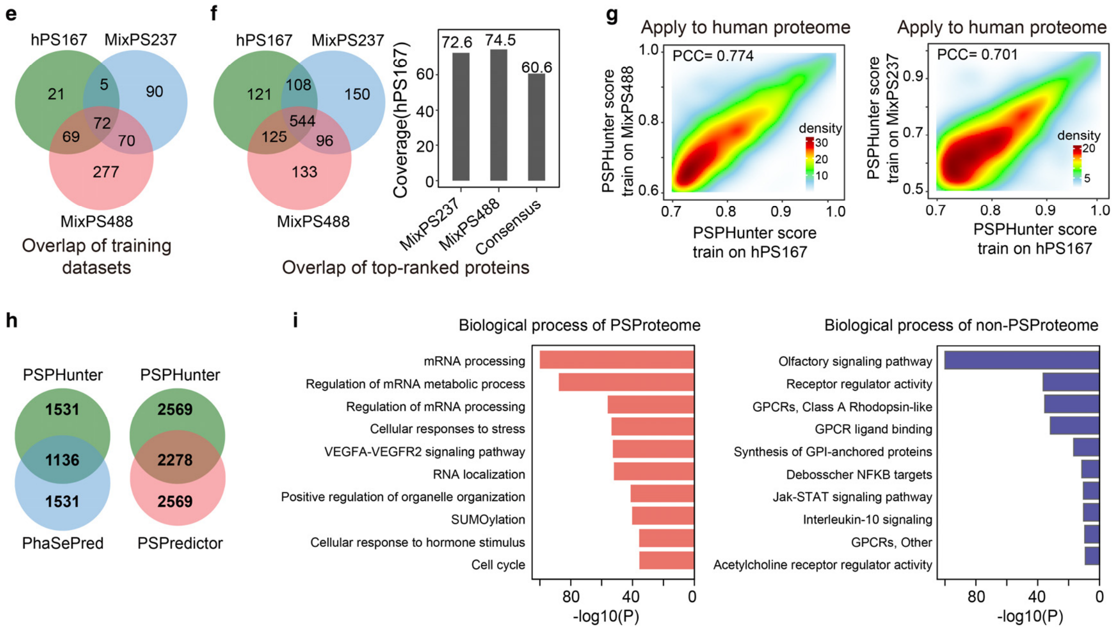

Supplementary Fig. 2 | Computational validation of potential phase-separating proteins. a, Data size with different redundancy. We employed blastclust, a subprogram of the blast software, to restrict sequence similarities among proteins. **b**, Violin plots comparing the performance among different datasets redundancies. Statistical significance was analyzed with one-sided Wilcoxon test, n.s., no significance, *P < 0.05, **P < 0.01, ***P < 0.001, ****P < 0.0001, n = 100 each. **c**, Violin plots comparing the performance among different ensemble strategies. AvgProb represents the averaged predicted probabilities from individual class-specific sub-classifiers, with classification performed based on the averaged probabilities. TierStacking refers to the two-layer stacking model, where the initial step involves obtaining predicted probabilities from each sub-classifier, followed by utilizing these probabilities as inputs for the subsequent modeling and classification. Statistical significance was analyzed with one-sided Wilcoxon test, ****P < 0.0001, n = 100 each. **d,** Comparison between

existing methods and PSPHunter, encompassing models, data resources, organisms, and features employed. **e,** Intersection of different training datasets. hPS167 represents the 167 phase-separating proteins used by PSPHunter, MixPS237 represents the PS-self proteins from PhaSePred (Chen et al., *PNAS*, 2022), MixPS488 represents a collection of scaffold phase-separating proteins sourced from the comprehensive review authored by Pancsa et al. **f,** Application of the three models to predict the entire proteome. We selected the top 898 proteins with the highest predicted phase separation probabilities and found that the MixPS237 model covers 72.6% of the proteins predicted by PSPHunter, the MixPS488 model covers 74.5%, and all three models together cover 60.6% of the proteins. **g,** Correlation between PSPHunter-predicted phase-separating probabilities and those predicted by the MixPS237 and MixPS488 models. **h**, Overlap between the PSProteome and the latest two phase separation predictors. In panel (g), proteins were ranked based on their PSPHunter scores, and an equivalent number of phase-separating proteins predicted by the PhaSePred and PSPredictor methods were selected for comparison. **i**, Functional analysis of PSProteome (left) and non-PSProteome (right) identified by PSPHunter.

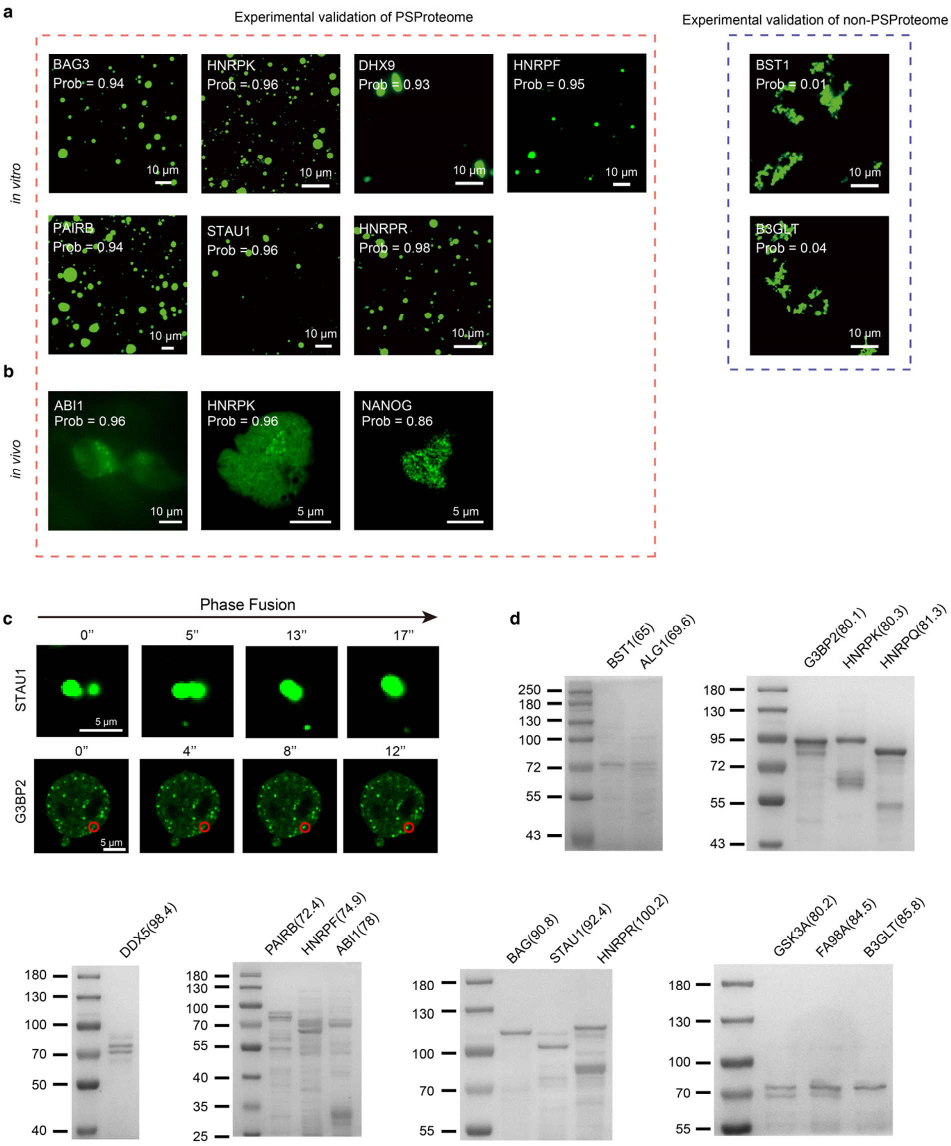

Supplementary Fig. 3 | Experimental validation of potential phase-separating proteins. a,b, Representative images showing green puncta formed by PSProteome and irregular overall shapes of condensates formed by non-PSProteome *in vitro* (a) and HEK293T cells (b). **c**, Droplet fusion behavior of STAU1 puncta *in vitro* and droplet fusion behavior of G3BP2 puncta in G3BP2-EGFP HEK293T cells. **d**, Coomassie brilliant blue staining showing the successful purification of potential phase-separating proteins.

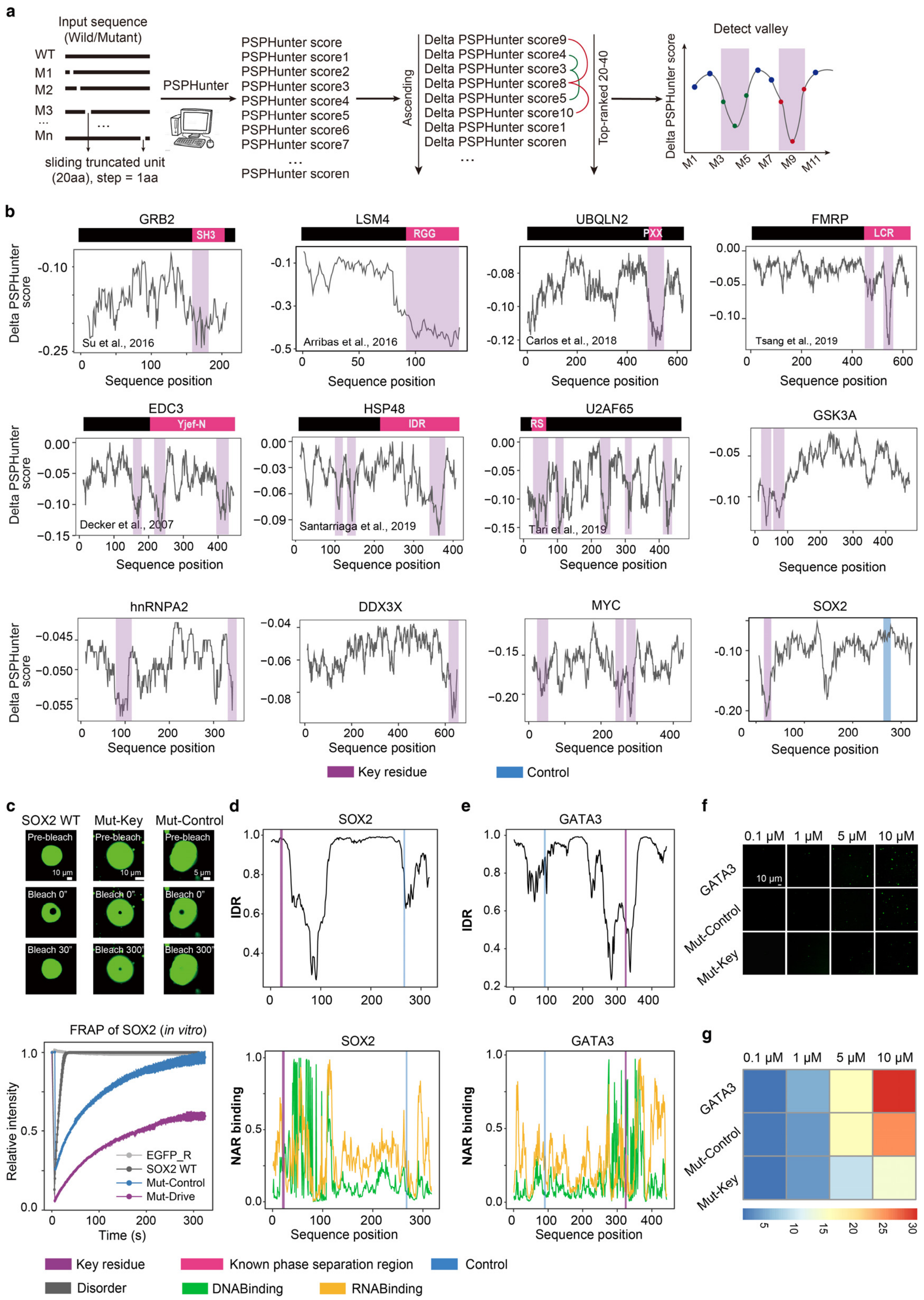

Supplementary Fig. 4 | Key regions of other typical phase-separating proteins. **a**, Detailed strategy diagram for identifying key residues. **b,** Predicted key residues of GRB2, LSM4, UBQLN2, FMRP, EDC3, HSP48, U2AF65, GSK3A, hnRNPA2, DDX3X, MYC and SOX2. The region filled in purple denoted the key region predicted by PSPHunter. The region filled in pink denoted known phase separation related region. **c**, FRAP analysis of SOX2-GFP. Representative imaging (top) and GFP fluorescence

intensity curve (bottom) showing the droplets of wild type and key residue-truncated SOX2 are rapidly recovered from photobleaching, while control residue-truncated SOX2 are hard recovered from photobleaching. **d-e**, Predicted key residues (purple), IDR (grey), DNA-binding region (green) and RNA-binding region (yellow) of SOX2 (d) and GATA3 (e). **f,** Representative images of GATA3 and its mutants at different concentrations. The purple region represents the truncation of key residues, which is expected to have a significant impact on phase separation. In contrast, the blue region denotes truncation of the control residues with minimal effect on phase separation. **g,** Quantification of puncta formation in GATA3 and its mutants at different concentrations.

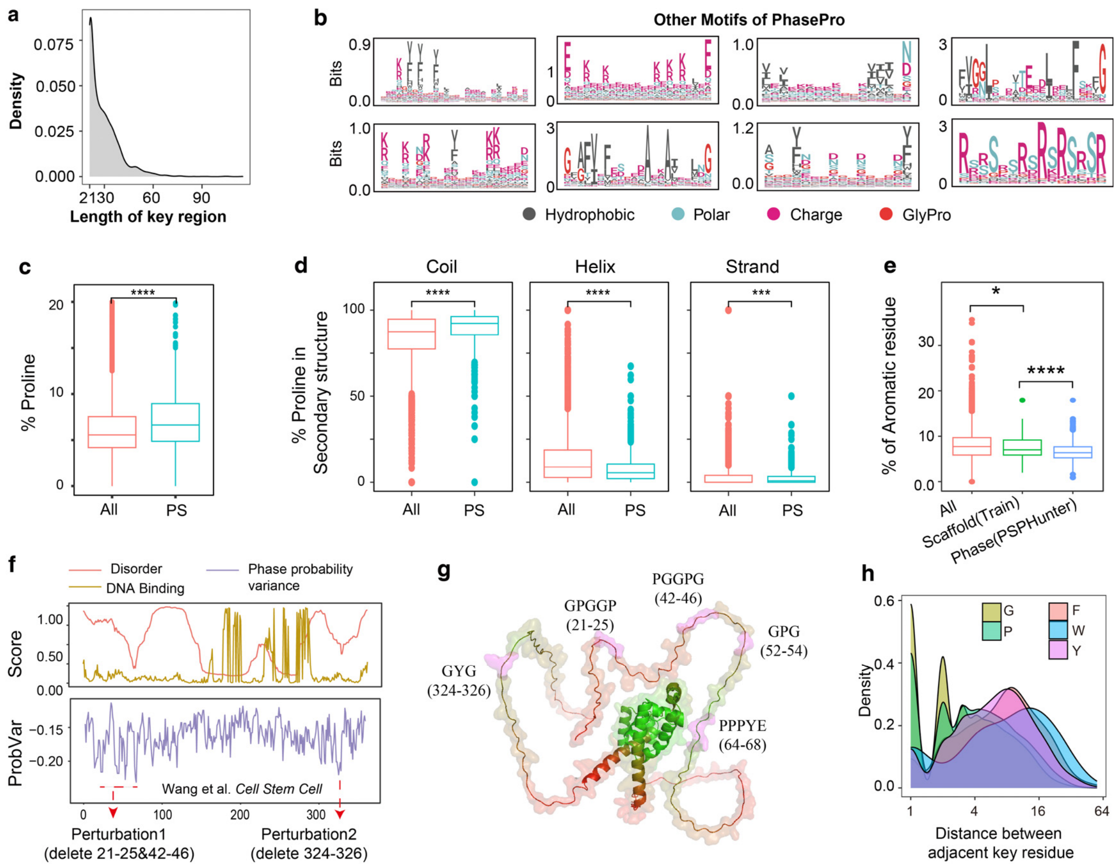

Supplementary Fig. 5 | Exploring characteristics of key residues for protein phase separation. a, The length distribution of key regions. **b**, Representative motifs of phase-separating proteins and key regions. **c,** Proportions of proline amino acids in all human proteins and phase-separating proteins. **d,** Proportions of proline amino acids in different secondary structures. The secondary structure predictions were generated using the SPIDER2 software. **e,** Proportions of aromatic amino acids in all human proteins, scaffold proteins in our training dataset and potential phase separation proteins predicted by PSPHunter. **f,** Key residues in OCT4. Notably, both segments of key residues in non-nucleic acid binding regions of the OCT4 protein, with these regions being enriched in proline residues. **g,** Representation of key residues in the structure of OCT4. The purple regions indicating the location of key residues. Proline amino acids are more inclined to be situated in the bend regions of intrinsically disordered regions. **h,** Distribution of sequence distances for specific amino acid types in key residues.

Note: All statistical tests were one-sided Wilcoxon tests. Significance levels are indicated by asterisks: *, $P < 0.05$; **, $P < 0.01$; ***, $P < 0.001$; ****, $P < 0.0001$ (not significant, denoted as n.s.). The error bars represent the standard deviation. The boxplots were drawn from lower quartile (Q1) to upper quartile (Q3), with the middle line denoting the median, and whiskers with maximum 1.5 interquartile range (IQR).

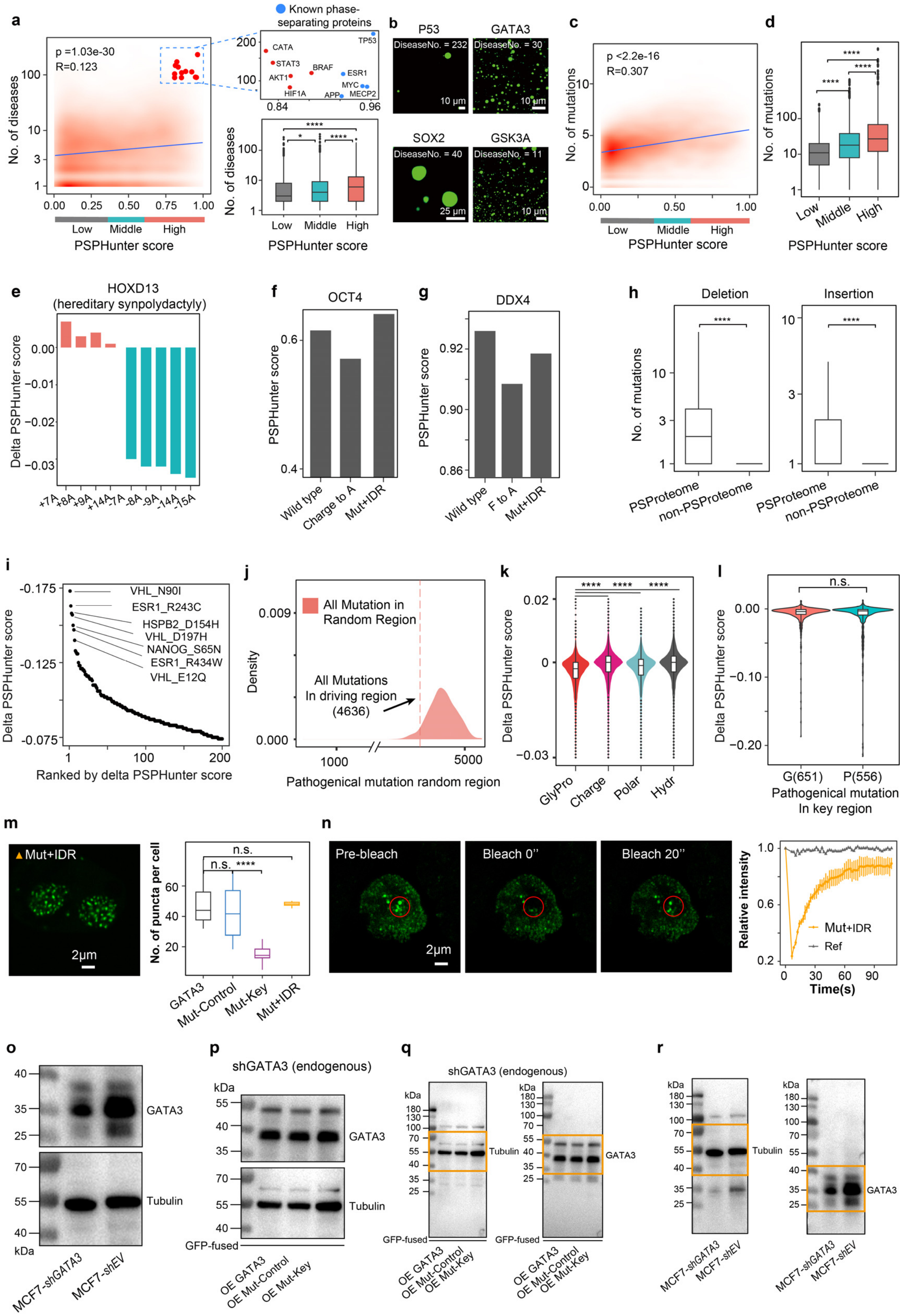

Supplementary Fig. 6 | The pathogenic mutations glycine and proline disrupt phase separation more significantly than other mutations. a, Heatmap showing positive correlation (Pearson correlation =0.123, *p*=1.03e-30) between a protein's phase separation capacity and the number of diseases in which it is implicated (one-sided Wilcoxon test, ****P < 0.0001; Low, n = 2184; Middle, n = 4332; High, n = 2170).

In this heatmap, red denotes proteins with relatively higher PSPHunter scores (greater than 0.61), cyan represents proteins with intermediate PSPHunter scores (ranging from 0.36 to 0.61), and grey designates proteins with lower PSPHunter scores (less than 0.36). Notably, proteins associated with the highest number of disease types are characterized as typical phase-separating proteins, including MYC, MECP2, P53, among others (highlighted in the top-right quadrant in red). **b**, Representative imaging of typical phase-separating proteins and the number of disease types involved. **c**, Heatmap showing positive correlation (Pearson correlation =0.307, p<2.2e-16) between a protein's phase separation capacity and the number of mutations. Red denotes proteins with relative higher PSPHunter score (greater than 0.61). Cyan denotes proteins with middle PSPHunter score (between 0.36 and 0.61). Grey denotes proteins with lower PSPHunter score (less than 0.36). **d,** Statistical analysis reveals that proteins with higher PSPHunter scores tend to harbor a greater number of mutations. **e,** Evaluating phase separation changes of protein HOXD13 in different mutation types. The alanine-rich region of HOXD13 will increase its phase separation ability by extending more alanine, and vice versa decrease its phase separation ability. **f-g**, Evaluating phase separation probability of protein OCT4(f) and DDX4(g) in massive residue mutations and peptide fusion. The peptide is the IDR region of the typical phase-separating protein FUS. **h**, Violin plots comparing the number of deletion and insertion mutations in the PSProteome and Non-PS (one-sided Wilcoxon test, ***P < 0.001, ****P < 0.0001). **i**, Pathogenic mutations that most affect phase separation capacity are detailed in this picture. **j**, Frequency distribution of mutations falling in random regions which have the equal length as the key region of the corresponding protein. The dashed line denoted the number of mutations that fall into the key regions. **k**, The impact of different types of pathogenic mutations in key residues on phase separation. Glycine and proline mutations (red), charge residue mutations (pink), polar residue mutations (cyan) and hydrophobic mutations (grey). Statistical significance was analyzed with one-sided Wilcoxon test, ****P < 0.0001. **l**, Violin plots show that the pathogenic mutations of glycine and proline have no difference in affecting phase separation capacity (Wilcoxon test, n.s., no significance). **m**, Representative imaging and *in vivo* quantification of puncta numbers for GATA3 and its mutants. The number of puncta of GATA3 per cell indicates that truncation of key residues significantly decreases the number of puncta, whereas fusion of IDR to GATA3 rescues its phase separation capacity(one-sided Wilcoxon test, ****P < 0.0001). **n**, FRAP analysis of Mut+IDR. Representative imaging (left) and GFP fluorescence intensity curve (right) demonstrate that the droplets formed by Mut+IDR rapidly recover from photobleaching. The error bars represent the standard deviation. **o**, Western blot shows that shGATA3 can significantly knock down the expression of GATA3. **p**, The expression of overexpressed GATA3 and its corresponding mutants was essentially the same in MCF7 cell lines that knocked out endogenous GATA3. **q,r** the original Western blot images corresponding Extended Data Fig 6 **o,p**.

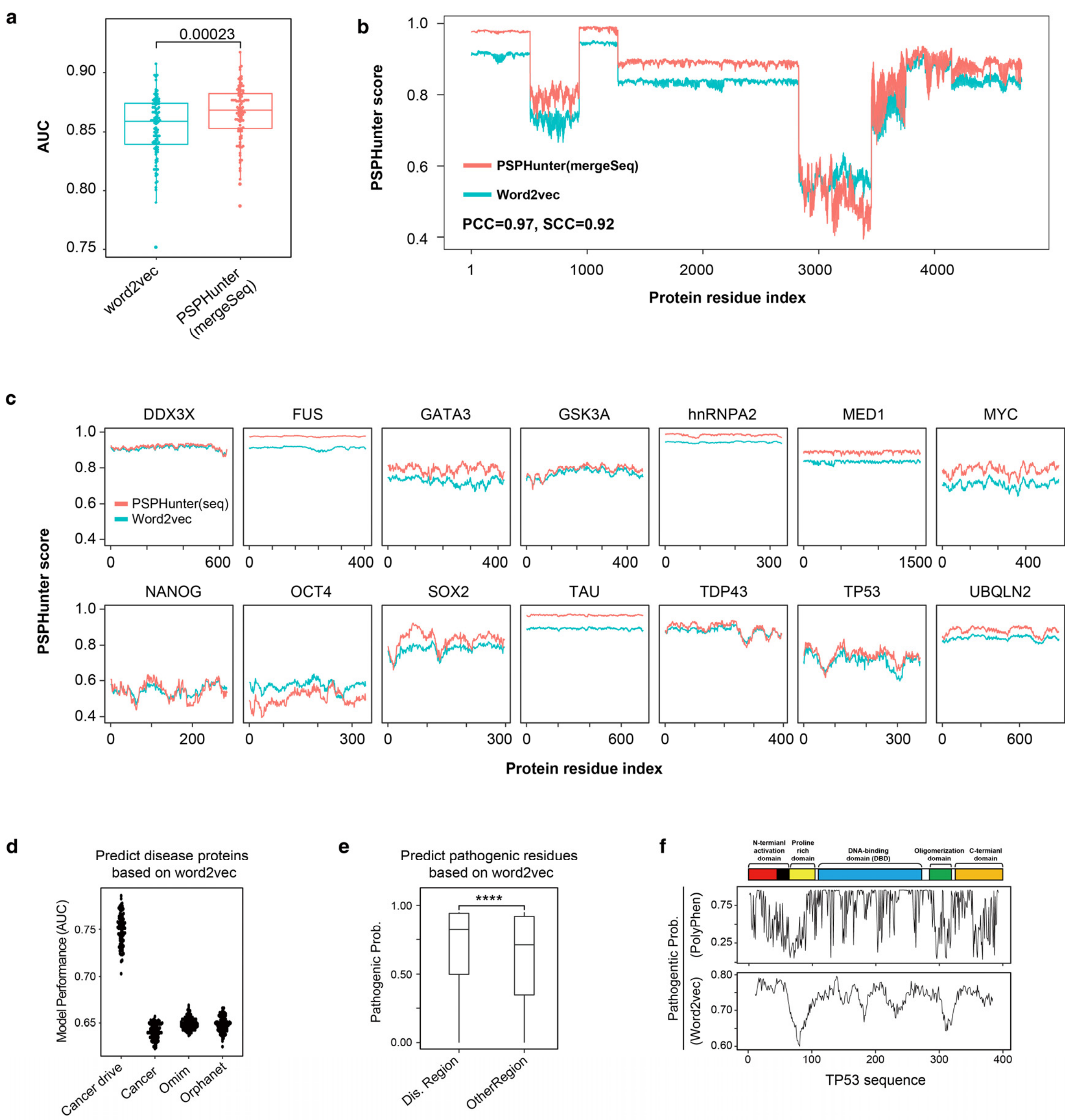

Supplementary Fig. 7 | The Word2vec descriptor is effective in the prediction of key residues and can also be applied to pathogenic residue prediction. a, Performance of word2vec model and PSPHunter(seq) model in prediction of phase separating proteins. **b**, Word2vec and PSPHunter(seq) show a high correlation in the prediction of key residues. **c**, Comparison of Word2vec model and PSPHunter(seq) in the prediction of key residues among the typical phase separating proteins. **d-f**, Technical advances of PSPHunter can be applied to pathogenic residue discovery (**d**) The descriptor word2vec is effective in identifying disease proteins, especially cancer key proteins (**e**) Our sliding-window truncation strategy was successfully applied to discover the pathogenic residues. We systematically identified the pathogenic residues of all cancer drive proteins. According to the pathogenic score of each residue predicted by the PolyPhen (Adzhubei et al., *Nature methods*, 2010, a tool for predicting the functional effect of human missense mutations), we illustrated that the pathogenic residues predicted by our algorithm have more impact on protein pathology (**f**) The distribution of pathogenic probabilities for the typical disease protein TP53 predicted by PolyPhen and word2vec, respectively. The lower the pathogenic score predicted by

word2vec, the greater the effect of the residues on the pathogenicity of the protein. We illustrated that the sliding-window truncation strategy can be used to discover the fine contribution of each residue to a specific protein attribute.

Note: All statistical tests were one-sided Wilcoxon tests. Significance levels are indicated by asterisks: *, P < 0.05; **, P < 0.01; ***, P < 0.001; ****, P < 0.0001 (not significant, denoted as n.s.). The boxplots were drawn from lower quartile (Q1) to upper quartile (Q3), with the middle line denoting the median, and whiskers with maximum 1.5 interquartile range (IQR).

Supplementary Table 1. Datasets used to construct PSPHunter.

Dataset	Data bank	Type	Description	Number of samples
Training_I (MixPS488)	Pancsa et al., *Briefings in Bioinformatics*, 2021	Mixed Species	Scaffold proteins and Negative samples	532
Training_II (MixPS237)	Chen et al., PNAS, 2022	Mixed Species	Self PS proteins and Negative samples	242
Training_III (hPS167)	PhaSepDB, LLPSDB, DrLLPS and PhaSePro	Human	Scaffold proteins and Negative samples	190
Independent_Test_I_MixPS488	Pancsa et al., *Briefings in Bioinformatics*, 2021	Mixed Species	Scaffold PS proteins and Negative samples	226
Independent_Test_II_MixPS237	Chen et al., PNAS, 2022	Mixed Species	Self PS proteins and Negative samples	102
Independent_Test_III_hPS167	PhaSepDB, LLPSDB, DrLLPS and PhaSePro	Human	Scaffold PS proteins and Negative samples	80
Independent_Test_IV_NonHuman_MixPS237	Pancsa et al., *Briefings in Bioinformatics*, 2021	Non-human	Self PS proteins and Negative samples	254
Independent_Test_V_NonHuman_MixPS488	Chen et al., PNAS, 2022	Non-human	Scaffold PS proteins and Negative samples	424

Supplementary Table 2. Feature Evaluation in Cross-Validation on Training_III (hPS167).

Model	Features	Recall	Precision	F1	ACC	MCC	AUC
Training_III (hPS167)	AAComposition	0.700	0.684	0.684	0.684	0.375	0.750
Training_III (hPS167)	EvoConservation	0.768	0.744	0.750	0.749	0.503	0.830
Training_III (hPS167)	PredFunctional site	0.730	0.742	0.729	0.734	0.476	0.816
Training_III (hPS167)	Word2vec	0.794	0.770	0.775	0.775	0.556	0.854
Training_III (hPS167)	MergeSeq	0.807	0.777	0.786	0.784	0.575	0.866
Training_III (hPS167)	Protein annotations	0.711	0.694	0.695	0.695	0.397	0.763
Training_III (hPS167)	Mut & PTM	0.733	0.760	0.740	0.748	0.502	0.833
Training_III (hPS167)	Network properties	0.765	0.823	0.787	0.798	0.602	0.861
Training_III (hPS167)	MergeFun	0.814	0.850	0.827	0.834	0.671	0.903
Training_III (hPS167)	MergeFeature	0.863	0.867	0.861	0.864	0.730	0.935

Supplementary Table 3. Feature Evaluation in Independent_Test_III_hPS167.

Model	Features	Recall	Precision	F1	ACC	MCC	AUC
Training_III (hPS167)	AAComposition	0.711	0.685	0.695	0.690	0.383	0.753
Training_III (hPS167)	EvoConservation	0.775	0.743	0.756	0.751	0.505	0.834
Training_III (hPS167)	PredFunctional site	0.751	0.745	0.746	0.745	0.493	0.822
Training_III (hPS167)	Word2vec	0.812	0.775	0.791	0.786	0.576	0.859
Training_III (hPS167)	MergeSeq	0.826	0.785	0.803	0.798	0.600	0.872
Training_III (hPS167)	Protein annotations	0.716	0.692	0.701	0.697	0.397	0.774
Training_III (hPS167)	Mut & PTM	0.726	0.758	0.739	0.745	0.494	0.829
Training_III (hPS167)	Network properties	0.781	0.822	0.799	0.804	0.611	0.865
Training_III (hPS167)	MergeFun	0.834	0.856	0.843	0.845	0.693	0.908
Training_III (hPS167)	MergeFeature	0.854	0.868	0.857	0.861	0.725	0.933

Supplementary Table 4. Assessment of Alternative Predictors on Independent_Test_III_hPS167.

Model	Dataset	Recall	Precision	F1	ACC	MCC	AUC
catGRANULE	Independent_Test_III_hPS167	0.863	0.656	0.744	0.706	0.437	0.803
CRAPome	Independent_Test_III_hPS167	0.537	0.910	0.673	0.744	0.533	0.784
DDX4	Independent_Test_III_hPS167	0.282	0.812	0.415	0.611	0.286	0.610
LARK	Independent_Test_III_hPS167	0.068	0.798	0.124	0.531	0.126	0.526
PLAAC	Independent_Test_III_hPS167	0.221	0.967	0.357	0.610	0.335	0.606
PScore	Independent_Test_III_hPS167	0.735	0.676	0.703	0.693	0.389	0.773
PSPred	Independent_Test_III_hPS167	0.673	0.754	0.710	0.736	0.474	0.792
RY	Independent_Test_III_hPS167	0.330	0.762	0.457	0.616	0.277	0.624
PSPHunter	Independent_Test_III_hPS167	0.854	0.868	0.857	0.861	0.725	0.933

Supplementary Table 5. Cross-Validation Performance Across Training_I, Training_II, and Training_III Datasets.

Model	Features	Recall	Precision	F1	ACC	MCC	AUC
Training_I (MixPS488)	MergeFeature	0.852	0.825	0.837	0.835	0.672	0.912
Training_II (MixPS237)	MergeFeature	0.851	0.829	0.836	0.836	0.675	0.907
Training_III (hPS167)	MergeFeature	0.863	0.867	0.861	0.864	0.730	0.935

Supplementary Table 6. Assessment of Independent_Test_IV and Independent_Test_V (Relate to Fig2a).

Model	Dataset	Recall	Precision	F1	ACC	MCC	AUC
Training_III (hPS167)	Independent_Test_IV_NonHuman_MixPS237	0.756	0.870	0.808	0.821	0.649	0.901
Training_III (hPS167)	Independent_Test_V_NonHuman_MixPS488	0.594	0.841	0.695	0.740	0.504	0.852
