# Supplementary material for: Precise prediction of phase-separation key residues by machine learning
Source: Nat Commun. 2024 Mar 26;15:2662. doi: 10.1038/s41467-024-46901-9 (PMC10965946; doi:10.1038/s41467-024-46901-9)
Supplement: Supplementary file 2 — Reporting Summary [file 41467_2024_46901_MOESM2_ESM.pdf]

nature portfolio

Corresponding author(s): Junjun Ding

Last updated by author(s): Mar 7, 2024

Reporting Summary

Nature Portfolio wishes to improve the reproducibility of the work that we publish. This form provides structure for consistency and transparency in reporting. For further information on Nature Portfolio policies, see our Editorial Policies and the Editorial Policy Checklist.

Statistics

For all statistical analyses, confirm that the following items are present in the figure legend, table legend, main text, or Methods section.

n/a	Confirmed	
☐	☒	The exact sample size (*n*) for each experimental group/condition, given as a discrete number and unit of measurement
☐	☒	A statement on whether measurements were taken from distinct samples or whether the same sample was measured repeatedly
☐	☒	The statistical test(s) used AND whether they are one- or two-sided<br>*Only common tests should be described solely by name; describe more complex techniques in the Methods section.*
☒	☐	A description of all covariates tested
☐	☒	A description of any assumptions or corrections, such as tests of normality and adjustment for multiple comparisons
☐	☒	A full description of the statistical parameters including central tendency (e.g. means) or other basic estimates (e.g. regression coefficient) AND variation (e.g. standard deviation) or associated estimates of uncertainty (e.g. confidence intervals)
☐	☒	For null hypothesis testing, the test statistic (e.g. *F*, *t*, *r*) with confidence intervals, effect sizes, degrees of freedom and *P* value noted<br>*Give P values as exact values whenever suitable.*
☒	☐	For Bayesian analysis, information on the choice of priors and Markov chain Monte Carlo settings
☒	☐	For hierarchical and complex designs, identification of the appropriate level for tests and full reporting of outcomes
☐	☒	Estimates of effect sizes (e.g. Cohen's *d*, Pearson's *r*), indicating how they were calculated

Our web collection on statistics for biologists contains articles on many of the points above.

Software and code

Policy information about availability of computer code

Data collection	The online Methods section provides detailed information. We gathered phase-separating proteins from the following databases: PhaSepDB (You, K. et al, 2020), LLPSDB (Li, Q. et al., 2020), DrLLPS (Ning, W. et al. 2020), and PhaSePro (Mészáros, B. et al. 2019). Post-modification data was extracted from the PhosphoSitePlus database (Hornbeck, P.V. et al. 2015), and mutation information was sourced from the HuVarBase database (Ganesan, K. et al., 2019).
Data analysis	The online Methods section provides detailed information. The parameters used for data analysis are comprehensively described, and if you require custom scripts, they are available upon request. The tools and software used in this study include PSI-BLAST (v 2.2.31+), HHblits (v 2.0.16), SPIDER2 (v 2.0.0), SPINE-D (v 2.0.0), SNBRfinder (v 1.0), GPS (v 5.0), Rhapsody (v 1.0), MEME (v 5.4.1), Gensim (https://radimrehurek.com/gensim/), scikit-learn (v 1.2.0), PhaSePred (http://predict.phasep.pro/), PSPredictor (http://www.pkumdl.cn/PSPredictor/), and Metascape (https://metascape.org/), MAGS115 (https://github.com/ekuec/2019_StressGranuleFeatures/). Additionally, you can access the custom scripts for analysis at https://github.com/jsun9003/PSPHunter.

For manuscripts utilizing custom algorithms or software that are central to the research but not yet described in published literature, software must be made available to editors and reviewers. We strongly encourage code deposition in a community repository (e.g. GitHub). See the Nature Portfolio guidelines for submitting code & software for further information.

Data

Policy information about availability of data

All manuscripts must include a data availability statement. This statement should provide the following information, where applicable:

- Accession codes, unique identifiers, or web links for publicly available datasets
- A description of any restrictions on data availability
- For clinical datasets or third party data, please ensure that the statement adheres to our policy

The pre-established model, along with the associated training and testing datasets of PSPHunter, and the predicted phase separation probabilities of all human proteins using PSPHunter, are available at https://github.com/jsun9003/PSPHunter. Uncropped scans of all blots and gels in Figures, along with the relevant raw data from each figure or table, are provided in the Supplementary Information/Source Data file.

Research involving human participants, their data, or biological material

Policy information about studies with human participants or human data. See also policy information about sex, gender (identity/presentation), and sexual orientation and race, ethnicity and racism.

Reporting on sex and gender	None
Reporting on race, ethnicity, or other socially relevant groupings	None
Population characteristics	None
Recruitment	None
Ethics oversight	None

Note that full information on the approval of the study protocol must also be provided in the manuscript.

Field-specific reporting

Please select the one below that is the best fit for your research. If you are not sure, read the appropriate sections before making your selection.

☒ Life sciences ☐ Behavioural & social sciences ☐ Ecological, evolutionary & environmental sciences

For a reference copy of the document with all sections, see nature.com/documents/nr-reporting-summary-flat.pdf

Life sciences study design

All studies must disclose on these points even when the disclosure is negative.

Sample size	No statistical method was utilized to predetermine the sample size for this study. Instead, sample sizes were explicitly indicated in the legends of each Figure and Supplementary Figures. The selection of sample sizes was made empirically, drawing upon common experience within the relevant fields. Our aim was to ensure that the chosen sample sizes provided sufficient statistical power to detect the indicated biological effects.
Data exclusions	No data are excluded from this study.
Replication	Where relevant, the number of biological replicates were provided in the figure legends. In vitro experiments were performed with three biological replicates to capture variability. All replication attempts were successful. The number of replicates was described in the figure legends and statistics section.
Randomization	There was no allocation of test subjects for any experiments, thus randomization is not relevant in this study.
Blinding	Quantifications were performed in a blinded manner by two or three scientists. Individuals analyzing the FRAP data were not aware of experimental groups until data collection was completed. All data analyses were performed by unbiased software programs/algorithms.

Reporting for specific materials, systems and methods

We require information from authors about some types of materials, experimental systems and methods used in many studies. Here, indicate whether each material, system or method listed is relevant to your study. If you are not sure if a list item applies to your research, read the appropriate section before selecting a response.

Materials & experimental systems

n/a	Involved in the study	
☐	☒	Antibodies
☐	☒	Eukaryotic cell lines
☒	☐	Palaeontology and archaeology
☒	☐	Animals and other organisms
☒	☐	Clinical data
☒	☐	Dual use research of concern
☒	☐	Plants

Methods

n/a	Involved in the study	
☒	☐	ChIP-seq
☒	☐	Flow cytometry
☒	☐	MRI-based neuroimaging

Antibodies

Antibodies used

The antibodies used for WB:
1. anti-GATA3, Rabbit, Monoclonal, ABclonal, Cat. number A19636, Lot number 4000000115 (1:1000)
2. anti-β-Tubulin, Rabbit, Polyclonal, ABclonal, Cat. number AC008, Lot number 3523022349 (1:200)

Validation

Antibodies were commercially validated as below.

1. anti-GATA3, Rabbit, Monoclonal, ABclonal, Cat. number A19636, Lot number 4000000115
Validation: https://abclonal.com.cn/catalog/A19636

2. anti-β-Tubulin, Rabbit, Polyclonal, ABclonal, Cat. number AC008, Lot number 3523022349
Validation: https://abclonal.com.cn/catalog/AC008

Eukaryotic cell lines

Policy information about cell lines and Sex and Gender in Research

Cell line source(s)

The MCF7 cell line was a gift from Dr. Hai Hu (Sun Yat-sen Memorial Hospital). The HEK293T cell line was kindly gifted by Dr. Jianlong Wang from Icahn School of Medicine at Mount Sinai.

Authentication

None of the cell lines used were authenticated.

Mycoplasma contamination

All lines tested negative for mycoplasma contamination.

Commonly misidentified lines
(See ICLAC register)

None

Plants

Seed stocks

None

Novel plant genotypes

None

Authentication

None
