# Supplementary material for: Precise prediction of phase-separation key residues by machine learning
Source: Nat Commun. 2024 Mar 26;15:2662. doi: 10.1038/s41467-024-46901-9 (PMC10965946; doi:10.1038/s41467-024-46901-9)
Supplement: Supplementary file 4 — Description of Additional Supplementary Files [file 41467_2024_46901_MOESM4_ESM.pdf]

Title: Supplementary Data 1

Description: Phase separation probabilities of all the reviewed human proteins. The table presents the phase separation probabilities predicted by the PSPHunter algorithm for all proteins. The data are also available on our web service (http://psphunter.stemcellding.org/dataset.php). The first column of the table contains the UniProt ID of each protein, the second column contains the Gene Name, the third column contains the PSPHunter score, and the fourth column indicates the protein type. In particular, "PSProteome" denotes proteins associated with phase separation where the PSPHunter score is greater than or equal to 0.82, while "non-PSProteome" represents non-phase-separating proteins (with the lowest PSPHunter score). The numbers of proteins in both PSProteome and non-PSProteome categories are equivalent, as depicted in Fig. 1h.

Title: Supplementary Data 2

Description: Overlap between known and predicted phase-separating key regions. Known phase-separating regions were sourced and validated from the PhaSePro database (https://phasepro.elte.hu). Additionally, we aligned the predicted start and end regions of key residues identified by PSPHunter with the known phase-separating regions, as depicted in Fig. 2b.

Title: Supplementary Data 3

Description: Mutations predicted most impact phase separation. We evaluated the effect of all mutations on phase separation, including both pathogenic and neutral mutations. Here, we showcase the top 200 mutations with the most pronounced effect on phase separation probability. Additionally, below the table, we also provide an assessment of the impact of GP mutations within key regions on phase separation.

Title: Supplementary Data 4

Description: Phase-separating proteins utilized for PSPHunter construction. The dataset is also accessible via our web service (http://psphunter.stemcellding.org/dataset.php).
